# COVID-19 infection: an overview on cytokine storm and related interventions

**DOI:** 10.1186/s12985-022-01814-1

**Published:** 2022-05-26

**Authors:** Soheila Montazersaheb, Seyed Mahdi Hosseiniyan Khatibi, Mohammad Saeid Hejazi, Vahideh Tarhriz, Afsaneh Farjami, Faramarz Ghasemian Sorbeni, Raheleh Farahzadi, Tohid Ghasemnejad

**Affiliations:** 1 Molecular Medicine Research Center, Tabriz University of Medical Sciences, Tabriz, 5166614731 Iran; 2 Kidney Research Center, Tabriz University of Medical Sciences, Tabriz, Iran; 3 Food and Drug Safety Research Center, Tabriz University of Medical Science, Tabriz, Iran; 4 Novin Medical Genetic Laboratory, Mazandaran University of Medical Science, Sari, Iran; 5 Hematology and Oncology Research Center, Tabriz University of Medical Sciences, Tabriz, 5166614731 Iran

**Keywords:** COVID-19, CRS, IL6, IL1, IL-17, TNF-α, ARDS

## Abstract

Coronavirus disease 2019 (COVID-19) caused by the severe acute respiratory syndrome coronavirus-2 (SARS-CoV-2) has posed a significant threat to global health. This virus affects the respiratory tract and usually leads to pneumonia in most patients and acute respiratory distress syndrome (ARDS) in 15% of cases. ARDS is one of the leading causes of death in patients with COVID-19 and is mainly triggered by elevated levels of pro-inflammatory cytokines, referred to as cytokine storm. Interleukins, such as interleukin-6 (1L-6), interleukin-1 (IL-1), interleukin-17 (IL-17), and tumor necrosis factor-alpha (TNF-α) play a very significant role in lung damage in ARDS patients through the impairments of the respiratory epithelium. Cytokine storm is defined as acute overproduction and uncontrolled release of pro-inflammatory markers, both locally and systemically. The eradication of COVID-19 is currently practically impossible, and there is no specific treatment for critically ill patients with COVID-19; however, suppressing the inflammatory response may be a possible strategy. In light of this, we review the efficacy of specific inhibitors of IL6, IL1, IL-17, and TNF-α for treating COVID-19-related infections to manage COVID-19 and improve the survival rate for patients suffering from severe conditions.

**Graphical Abstract:**

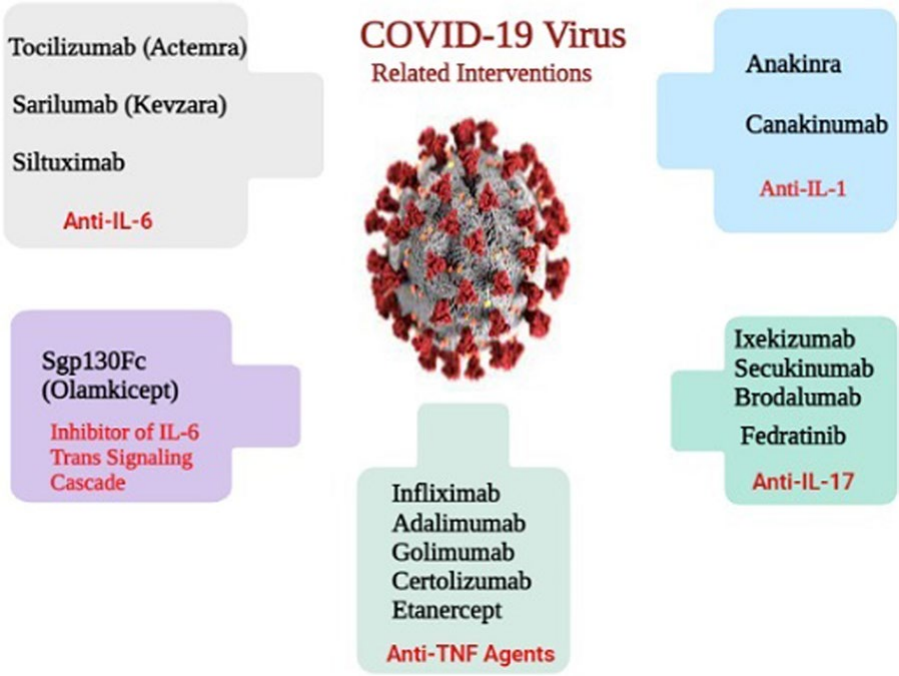

## Background

At the end of 2019, the severe acute respiratory syndrome coronavirus 2 (SARS-CoV-2) originated in Wuhan, China. Since then, it has been rapidly spreading worldwide and becoming a public health concern worldwide. This disease leads to pneumonia infection named coronavirus disease 2019 (COVID-19), posing an enormous threat to global health [1]. COVID-19 can trigger a cytokine storm in pulmonary tissues through hyperactivation of the immune system and the uncontrolled release of cytokines [2]. The phrase “cytokine storm” is a descriptive term to encompass a variety of events that may ultimately result in multi-organ failure and death [3]. Cytokine storms can cause a severe clinical complication known as acute respiratory distress syndrome (ARDS). ARDS is induced by an excessive immune response rather than the viral load [4]. Growing evidence is being documented on the possible role of the pro-inflammatory cytokines in COVID-19 pathogenesis and related complications [5]**.** With such a reason in mind, management of the cytokine release syndrome (CRS) and preventing subsequent infections may be an intriguing approach for COVID-19 therapy. Accordingly, there is an imperative need to develop anti-inflammatory agents for COVID-19 patients who develop CRS. Identifying the underlying mechanisms can aid in developing therapeutic strategies and speed up recovery. Several clinical trials are ongoing to investigate novel supportive care and interventions to cure this infection. To date, no definite and effective therapeutic agents are available. However, some supportive therapeutic interventions and anti-viral agents with limited efficacy are in hand to decrease the outbreak of COVID-19 and mitigate its symptoms [6, 7]. In this regard, anti-inflammatory therapy in patients with COVID-19 may be a promising intervention for COVID-19-related pneumonia [8]. This article will address the current understanding of cytokines-induced alterations in COVID-19, focusing on interleukin (IL)-6, IL-1, IL-17, and tumor necrosis factor (TNF). Then, we will discuss therapeutic strategies to rescue affected patients with severe COVID-19.

## Cytokine release syndrome

It is believed that the severity of COVID-19 disease is linked to the virus-induced cytopathic effects and escape of the virus from the host immune system [9]. In patients suffering from COVID-19, the host immune system can cause a lethal inflammatory situation known as CRS [10]. As the name reveals, this is a phenomenon of an extreme inflammatory response, in which inflammatory cytokines are rapidly secreted in a massive amount in response to infective stimuli. Indeed, this unconstrained inflammatory cytokine storm is a severe status observed in patients requiring intensive care unit (ICU) admission [11]. CRS is one of the possible events for the progressive and severe forms of COVID-19 and its mortality. Defining clinical criteria for CRS is a challenging issue; however current studies propose a series of features such as clinical symptoms and laboratory findings to confirm this status [12, 13]. The underlying mechanisms responsible for the unrestrained release of inflammatory factors are still vague, but several hypotheses exist. The first one is related to virus replication, which leads to pyroptosis, a highly inflammatory form of lytic-programmed cell death (apoptosis). In COVID-19 patients, pyroptosis triggers the release of pro-inflammatory cytokines and affects macrophage and lymphocyte functions [14, 15], causing peripheral lymphopenia [16]. Growing evidence points to an alteration in innate immunity induced by interferon (INF)-1. INF-1 is a vital contributor to viral replication and promoting the adaptive immune systems. Indeed, COVID-19 influences the host’s innate immune response and weakens the function of INF-1 in response to infection [14, 17]. Following virus infection, macrophages, dendritic cells, and neutrophils start the immune response as the body's first-line defense. Consistent with this notion, the lung autopsies from patients who died of COVID-19 revealed a high infiltration of macrophages into the bronchial mucosa [18]. Besides, recent studies suggest that excessive production of some cytokines, such as IL-6 may be the leading cause of inflammatory response in COVID-19 [19]. The second hypothesis is associated with adaptive immunity and the production of neutralizing antibodies against the surface antigen of the virus. Several animal studies declared that immunoglobulin (Ig) Gs could bind to the S protein and trigger inflammatory cascades. This binding can accumulate pro-inflammatory macrophages and monocytes in the lungs through the release of IL-8 and monocyte chemoattractant protein (MCP)-1. The inflammatory reaction is mediated by the Fc receptor (FcR) interaction on the surface of monocytes/macrophages with the virus-anti-S-IgG complex. This notion is supported by the decreased level of pro-inflammatory cytokine following blockage of macrophage receptors [20, 21]. In addition, several pieces of evidence support that anti-viral IgGs are coincident with the onset of severe respiratory disease in COVID-19 patients [22].

As mentioned earlier, fatalities in the severe form of COVID-19 are strongly associated with CRS. Virtually all cells and tissues in the body can be influenced by cytokine storms. CRS is an acute and uncontrolled inflammatory response characterized by multi-organ dysfunction and diverse clinical manifestations such as fever [3]. With these backgrounds, a vast range of anti-inflammatory approaches is being developed to dampen CRS and save the life of affected patients.

## COVID-19 and organ damage

Several pro-inflammatory cytokines can induce cell death in various cell types, leading to pathological conditions. In a study conducted on 416 patients affected with COVID-19, cardiac injury was present in about 19.7% during hospitalization of the patients [23]. There is a possibility that COVID-19 causes myocardial cell injury either directly by interacting with the angiotensin-converting enzyme-2 (ACE2) receptors or indirectly by other mechanisms [24].

In the case of ACE2 receptors, it is hypothesized that the COVID-19 first attacks several organs expressing ACE2 receptors, such as the heart, brain, vessels, liver, kidney, and, more importantly, lung [25]. Several lines of evidence declare that COVID-19 may cause brain injury via direct and indirect mechanisms. In this case, creating a hypercoagulable state leads to the occlusion of cerebral vessels and brain damage. Besides, cytokines may have neurotoxic effects on the brain and can disrupt the integrity of the blood–brain barrier (BBB), resulting in neurological manifestations [26]. These clinical effects are more common in patients with severe forms of COVID-19 disease [27].

The liver is another organ influenced by the cytokine storm of COVID-19. It has been reported that 14.8–53.1% of patients with COVID-19 had abnormal levels of serum aminotransferases [28]. Acute kidney injury is also prevalent in ICU admitted COVID-19 patients due to the activation of the innate and adaptive immune systems and inflammation caused by virus infiltration and cytopathic effects [29].

Importantly, ARDS seems to be the most serious complication of COVID-19, with a high mortality rate. In other words, ARDS is a consequence of CRS and leads to respiratory epithelium damage [30]. In these patients, the aberrant pathogenic T cells secrete higher levels of cytokines such as granulocyte–macrophage colony-stimulating factor (GM-CSF), resulting in an inflammatory storm and severe lung damage [31]. Relying on this concept and according to data from a meta-analysis of 38 studies involving 3062 patients with COVID-19, the incidence of ARDS cases was 19.5% and the fatality rate was 5.5% [32]. In addition to those mentioned above, cytokines have a critical role in other complications of COVID-19; for instance, IL-6 can reach the skin and induce skin lesions [33]. Collectively, the uncontrolled release of cytokines may cause multi-organ damage in COVID-19 patients.

## ACE2 receptors

**Fig. 1:**
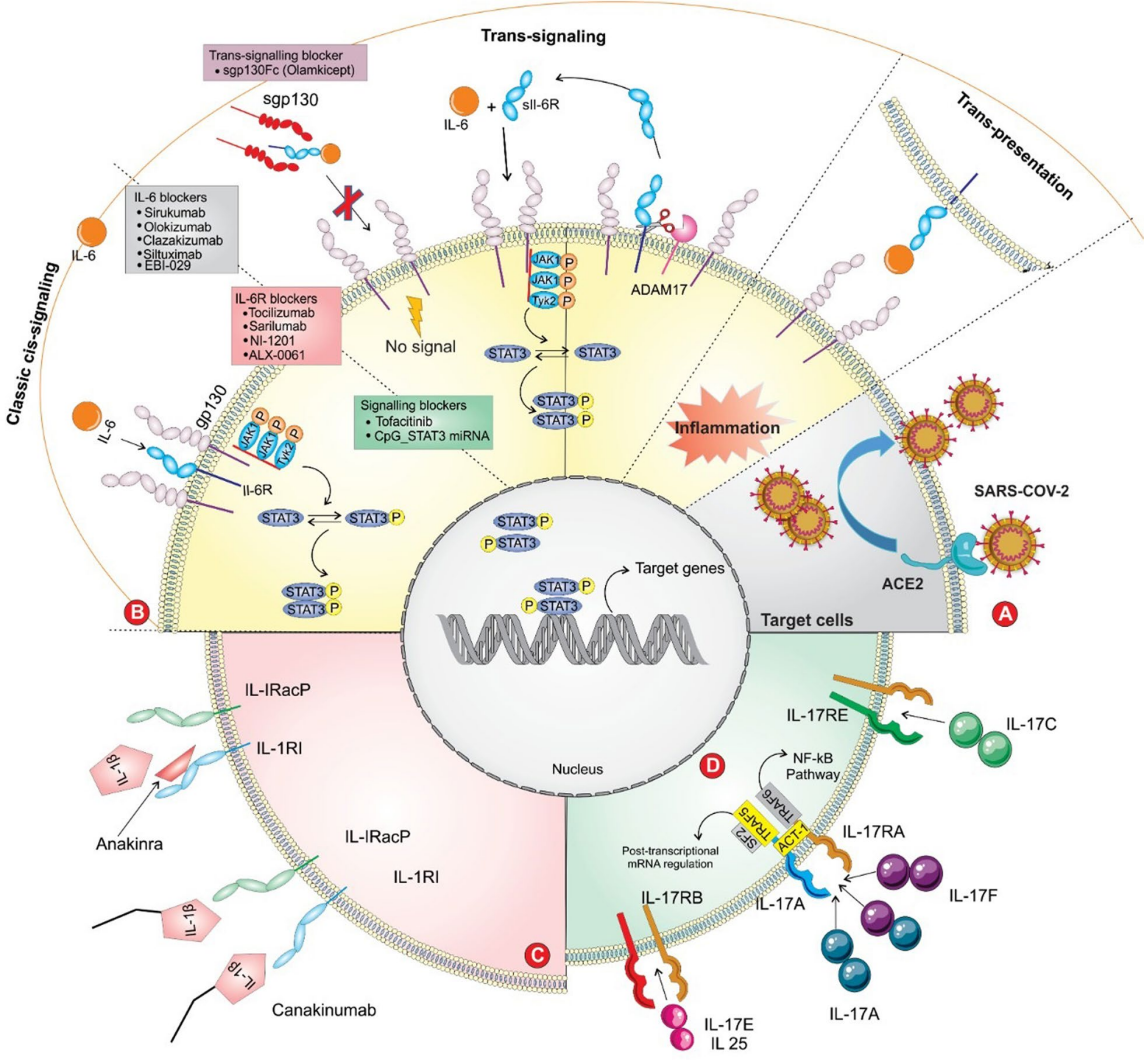
A panoramic review of IL-6, IL-1, IL-17, and related interventions in COVID-19-induced CRS. **A** The entry of SARS‐CoV‐2 into the ACE2‐expressing cells. **B** The modes of IL-6 signaling are depicted. IL-6 binds to the soluble or membrane-bound receptor, forming a complex with ubiquitously expressed gp130 protein. The intracellular domain of gp130 activates JAK/STAT signal transduction. The soluble form of IL-6R is mediated by the cleavage of ADAM17 enzyme. Antagonists of IL-6 (tocilizumab, sarilumab, and siltuximab) antagonize ligand-receptor engagement; thereby inhibiting IL-6 mediated signaling. sgp130Fc is an exclusive inhibitor of IL-6 trans-signaling. **C** Anakinra and Canakinumab antagonize the IL-1 mediated inflammation via binding to corresponding receptors. **D** IL-17 is a member of pro-inflammatory cytokines, having a critical role in the recruitment of monocytes and neutrophils to the inflamed sites. IL-17 has mediated its activity via binding to corresponding receptors (IL-17R), activating inflammation-related signaling. SARS-CoV-2: severe acute respiratory syndrome coronavirus-2; ACE2: angiotensin-converting enzyme 2; sIL-6R: the soluble form of the receptor; JAK/STAT: Janus kinase/signal transducer and activator of transcription

ACE2 receptors are the main binding site for virus entry and subsequent viral replication (Fig. 1A). ACE2, as a transmembrane receptor, is mainly found in type II alveolar cells, bronchial epithelial cells, myocardial cells, oesophagus epithelial cells, liver cholangiocytes, neurons and glia, stomach, cholangiocytes, adipose tissue, pancreatic exocrine glands and islets, renal tubules, stomach epithelial cells, ileum, rectum and many other sites [34]. In this regard, Zou et al. provided evidence regarding the potential risk of different organs to COVID-19 infection. Some tissues are more vulnerable to COVID-19 due to higher expression of ACE2, such as the lower respiratory tract, lung, heart, ileum, oesophagus, kidney, and bladder. Since the liver and stomach have lower levels of ACE2-positive cells, they are at low risk for COVID-19 infection [35].

## T cells in COVID-19 patients

Following COVID-19 infection, patients experienced exuberant activation of T cells. T helper (Th) cells have a critical role in the adaptive immune system in viral infections. In this context, type 1 interferon production is inefficient and anti-viral response is impaired. Indeed, Th1 cells can mainly regulate the adaptive immune system via cytokine production, while cytotoxic T-lymphocytes (known as CD8^+^ T cells) act as specific mediators to destroy virus-infected cells. Converging evidence has revealed that COVID-19 infection affects T lymphocytes and decreases the numbers of CD4^+^ and CD8^+^ T cells and interferon-gamma (IFN-γ) levels. It is crucial to note that the reduction of T cells is correlated with the severity of COVID-19 in affected patients. In this condition, Th_2_ responses are promoted and accompanied by overexpression of Th_2_-derived cytokines such as IL-4, IL-5, and IL-13 [36]. It is noteworthy to highlight that an effective immune response against viral-mediated infections relies on the activation of cytotoxic T cells to clear virus-infected cells. As a result, promoting the number and function of T cells is a crucial point for successful recovery in affected patients [37].

Moreover, a recent study pointed out that 82.1% of COVID-19 patients had a low circulating lymphocyte count [38]. Similarly, in a retrospective study by Diao and colleagues from data of 522 COVID-19 patients, 499 cases were analyzed for lymphocyte count. The results disclosed that 75.75%, 75.95%, and 71.54% of ICU admitted patients had low levels in the count of total T (lower than 800/μL), CD4^+^ (lower than 300/μL), and CD8^+^ T (lower than 400/μL) cells, respectively. These findings were negatively associated with the survival rate in infected patients. Apart from reducing the T-cells number, T cells were functionally exhausted. In addition, a negative correlation was observed between T cell counts and cytokine levels of serum IL-10, IL-6, and TNF-α. However, it should be noted that ACE2, as a defined receptor of the COVID-19 virus, is absent on T cells (11), proposing that the reduced level of T counts in affected patients is likely not resulting from direct infection of T cells [39].

## The role of inflammatory cytokines in CRS

Based on recent studies, it was strikingly shown that the level of inflammatory cytokines is increased in COVID-19. An overview of the literature indicates that IL-6, IL-2, IL-7, IL-10, granulocyte colony-stimulating factor (G-CSF), IFN- γ, inducible protein (IP)-10, TNF-α, MCP-1, macrophage inflammatory protein (MIP)-1α play a crucial role in the pathogenesis of COVID-19 [40]. Besides, Liu et al. (2015) evaluated 48 cytokines in the blood plasma of COVID-19 patients. Compared to healthy subjects, 38 out of 48 cytokines were remarkably elevated in patients with COVID-19. In addition, there was a strong linear association between severe lung injury and the level of 15 cytokines including, IFN-γ, IFN-α2, IL-1ra, IL-2, 4, 7, 10, 12, and 17, as well as chemokines such as IP-10, macrophage colony-stimulating factor (M-CSF) and G-CSF. The levels of Th1, Th2, and Th17 cells were increased, too [41].

With this evidence, a great deal of attention has been paid to dampening signaling pathways of inflammatory cytokines aiming to reduce inflammatory responses and mortality in patients suffering from COVID-19. According to the literature, cytokines have a key role in regulating immunological and inflammatory profiles. Among the cytokines, IL-6 is known as a causative factor in the pathogenesis and severity of COVID-19 due to various pleiotropic functions. Therefore, continuous measurement of IL-6 level is suggested in affected subjects with COVID-19. Multiple clinical trials are ongoing to evaluate the benefit of cytokine blockade by corresponding inhibitors. Taken together, we concisely describe inflammatory markers responsible for CRS and possible therapeutic approaches in this regard.

## The role of IL-6 signaling in inflammatory status

IL-6 is a glycoprotein that can act as both pro-inflammatory and anti-inflammatory cytokines. It can be produced by stromal, almost all immune cells, and other cells such as endothelial cells, fibroblasts, keratinocytes, and tumor cells. It has been well established that IL-6 has a crucial role in the differentiation of B-cells and the production of antibodies. Other immunomodulatory roles of IL-6 are linked to the development of self-reactive pro-inflammatory CD4^+^ T-cell response, stimulation of cytotoxic T-lymphocyte activity, regulation of T-helper 17, and regulatory T-cell balance [42, 43]. Besides, some evidence instantiates more effects of IL-6 on immune and non-immune cells through acting in a hormone-like fashion. In addition to this, IL-6 plays a critical regulatory role in homeostasis, such as the acute-phase response and hematopoiesis [44].

Growing studies have indicated that elevated levels of IL-6 are associated with CRS [45]. In other words, the pathophysiological hallmark of COVID is closely associated with severe inflammatory responses; thereby, identifying the serum level of IL-6 may predict the progression of COVID-19 disease. According to a systematic review and meta-analyses of 10 cohort studies, including 1798 patients, elevated levels of IL6 were observed in patients with COVID-19 [46]. One study showed that the serum level of IL-6 went up to 517 ± 796 pg/mL in patients with severe acute respiratory syndrome. In contrast, in recovered patients, the level of this cytokine gradually decreases to 68.8 ± 25.9 pg/mL [47]. Another similar research found that the level of IL-6 was noticeably higher in 86.8% of hospitalized COVID-19 individuals with severe complications and 22.9% had more than a tenfold increment in IL-6 levels [45].

IL-6 can signal primarily through cis- and trans-signaling pathways, which is distinct in cellular distribution (Fig. 1B) [48]. IL-6 first binds to its receptor (IL-6R), and then the IL-6/sIL-6R complex associates with the signal-transducing membrane protein gp130 to initiate intracellular signaling. The gp130 is expressed by most cells in the body, while IL-6R expression is mainly restricted to immune cells such as neutrophils, hepatocytes, monocytes-macrophages, and specific lymphocytes [49]. In the era of molecular-based analysis, in the cis-activating pathway, IL-6 binds to the membrane-bound IL-6 receptor (mbIL-6R, which express in immune cells), building a complex. This signaling pathway can predominate at a lower level of IL-6, thereby mediating the anti-inflammatory effects [50]. In other words, upon binding IL-6 to its receptor, the complex interacts with gp130, resulting in dimerization and activation of the Janus kinase/signal transducer and activator of transcription (JAK/STAT) pathway. Due to the broad expression of gp130 in various effector cells, the higher level of IL-6 is associated with strong hyperactivity of the immune system. Relying on this, cis-activation of IL-6 causes several effects on immune systems in terms of the acquired (B and T cells) and innate (macrophages, neutrophils, and natural killer cells) systems, developing CRS [51]. The consequence of JAK/STAT3 activation leads to a systemic CRS and releases a variety of cytokines such as IL-8, IL-6, vascular endothelial growth factor (VEGF), MCP-1, and E-cadherin. Both VEGF and E-cadherin enhance the permeability and leakage of vascular endothelial cells, resulting in pathophysiological disturbances and lung dysfunction/failure [52].

On the contrary, the soluble form of the receptor (sIL-6R) is found in serum and synovial fluids and mediates the trans-signaling cascade. This provides stimulation of a variety of cells by IL-6. The soluble form of IL-6R is generated from mbIL-6R cleavage by a disintegrin and metalloprotease 17 (ADAM17) [53]. In a healthy status, classical (cis) signaling has a protective role and controls various metabolic processes. IL-6 trans-signaling pathway is positively correlated with disease-induced inflammation. Moreover, inducing the differentiation of monocytes into macrophages, recruiting immune cells, and restraining Treg cell activity are mediated by IL-6 trans-signaling pathway [54].

In addition to mentioned signaling pathways, a third mode called trans-presentation is present for IL-6 signaling (Fig. 1B). This is a juxtacrine signaling pathway in which dendritic cells and T cells are involved in IL-6 signaling and induce various events, including the generation of pathogenic Th17 cells, Treg cells suppression by IL-27, and priming Th17 cells in combination with transforming growth factor-beta 2 (TGF-β2). Following this process, phosphorylation and activation of the JAK/STAT3 pathway occurred [55]. Furthermore, other pathways such as rat sarcoma-rapidly accelerated fibrosarcoma (RAS-RAF) and phosphatidylinositol 3-kinase/protein kinase B (PI3KB) can be activated by IL-6-mediated effects [56].

Recent evidence has implied the possible role of the inflammatory cytokines in the pathology of severe COVID-19 [31]. Given the essential role of IL-6 as a key driver in inflammatory status, and based on international guidelines, finding a suitable and efficient approach to inhibit IL-6 signaling is in demand. Accordingly, applying neutralizing antibodies to target IL-6- mediated inflammatory responses may be a life-saving strategy for COVID-19 patients. Numerous investigations have been performed to alleviate IL-6-mediated inflammation, mainly in the advanced stage of the disease. Two main types of IL-6 inhibitors as monoclonal antibodies are available, targeting IL-6 (siltuximab) or the corresponding receptor (tocilizumab and sarilumab).

## Application of monoclonal antibodies targeting IL-6 and IL-6R

### Tocilizumab (Actemra)

Tocilizumab (TCZ) is a recombinant humanized anti-IL-6R monoclonal antibody that can inhibit cytokine storms through blockade of IL-6 signaling (Fig. 1B). TCZ, as a food and drug administration (FDA)-approved drug, has exhibited effectiveness in the treatment of autoimmune diseases and various inflammatory conditions such as rheumatoid arthritis (RA), systemic juvenile idiopathic arthritis, neuromyelitis optica, and giant cell arteritis [57]. In addition, due to the pronounced inhibitory effects of TCZ on the inflammatory status, it can be used to treat chimeric antigen receptor T-cell-induced CRS [58]. Indeed, TCZ as a competitive antagonist for soluble and membrane IL-6R, inhibits both cis- and trans-signaling pathways, thus reducing inflammatory responses [59]. IL-6R inhibitors have been shown to have a high level of safety and efficacy in clinical studies compared with IL-6 inhibitors [60]. Considering the pivotal role of IL-6 in COVID-19 pathogenesis and CRS condition, reducing the level of IL-6 can improve the clinical outcome in patients with COVID-19. Hence there is a great interest in anti-IL-6 application in patients with severe lung problems and the elevated level of IL-6. Given the swift outbreak of this disease, China National Health Commission has quickly approved tocilizumab for the treatment of critical patients affected by COVID-19 [61].

Growing evidence has demonstrated the efficacy of tocilizumab therapy in COVID patients [62]. A study was conducted on 239 patients with COVID-19 and CRS, and 104 patients experienced worse conditions. All patients were administered tocilizumab. The results revealed that the survival rate was 83% in patients suffering from severe disease and 91% in patients with non-severe disease, indicating the potential role of tocilizumab in improving clinical outcomes [63].

In a retrospective study, Xu et al. (2020) showed that in addition to standard care therapy, administration of TCZ (400 mg) in 21 patients with severe COVID-19 patients resulted in 90% recovery, implying the effectiveness of the treatment [64]. In a prospective study in Italy on 100 patients with severe COVID-19, two-dose administration of TCZ every 12 h could improve the respiratory condition in a significant manner in 77 patients within 10 days. Although 23 of those 100 patients had dysfunction in the respiratory system and among them, 20 patients died [65]. However, another retrospective case–control survival analysis reported that TCZ had no efficacy in severe COVID-19 patients. Their results showed that the mortality rate was nearly similar between tocilizumab-treated and the control group who received standard care [66].

### Sarilumab (Kevzara)

Sarilumab is a recombinant human IL-6Rα antagonist that inhibits signal transduction by binding to both soluble and mbIL-6R, inhibiting classical and trans-signaling pathways (Fig. 1B). Sarilumab, like tocilizumab, is used to treat RA and has recently been administered to a series of patients suffering from severe COVID-19 and respiratory failure [67].

Numerous studies have assessed the tolerability and efficacy of sarilumab in patients suffering from RA. The data from a meta-analysis of four randomized controlled trials (RCTs) involving 2667 RA patients showed that sarilumab at 150 and 200 mg doses had efficacy and were well-tolerated [68]. A recent study showed the effectiveness of sarilumab in improving the symptoms of respiratory functions by decreasing oxygen demand by up to 30% [45]. Notably. the early intervention of sarilumab in patients with COVID-19 led to their discharge after 14 days of hospitalization, indicating the drug's efficacy in alleviating their symptoms [69].

Moreover, IL-6 showed the ability to down-regulate drug-metabolizing enzymes and corresponding transporters via binding to the cognate receptors. The application of anti-IL-6 agents can up-regulate the drug-metabolizing enzymes and transporters and reduce cytochrome P450 3A4 (CYP3A4) level [70], therefore drugs that are metabolized through CYP3A4 should not be administered by sarilumab. According to our overview of the published data, sarilumab can decrease the serum C-reactive protein (CRP) [71]. Surprisingly, after sarilumab administration, an increase in serum levels of IL-6 levels was observed in COVID patients. This can be due to the blockade of IL-6R and the reduction of IL-6 clearance. In this situation, no signaling has occurred even at a high level of IL-6 [72].

However, François-Xavier Lescure et al. randomly assigned 420 COVID-19 patients to receive either sarilumab (200 and 400 mg) or a placebo. On day 29, this placebo-controlled trial revealed no significant differences in survival rate between sarilumab receiving patients and placebo one, implying the ineffectiveness of sarilumab in severe conditions [73]. Despite all known efficacy of sarilumab, some adverse activity has been reported following administration of sarilumab, including hypersensitivity to some formulation components, viral reactivation, neutropenia, thrombocytopenia, elevated level of transaminase, and hyperlipidemia [74].

### Siltuximab

Siltuximab is a chimeric monoclonal antibody against human IL-6 (Fig. 1B). This is an FDA-approved drug used to treat patients suffering from multicentric castleman disease (MCD) [75]. Siltuximab binds to soluble IL-6 and prevents it from binding to corresponding receptors, inhibiting IL-6 signaling pathways [76]. According to the results of 21 patients in COVID-19 with ARDS who received siltuximab intravenously at doses between 700 and 1200 mg, CRP levels were significantly reduced in most patients. Besides, 33% of patients showed improvement in their clinical condition, 43% remained in a stable state with no clinical changes, and the condition of 24% of patients worsened [77].

In another study, Gritti et al. surveyed the effect of siltuximab treatment on serum cytokines and respiratory function in 21 hospitalized COVID-19 patients who developed ARDS. Siltuximab was intravenously administered at a median dose of 900 mg. Among patients, five received a second dose. Following treatment, CRP levels reached the normal value by day 5 in 16 patients. Additionally, there was a significant improvement in ventilation performance in 7 patients, and 43% of patients had stabilized clinical conditions [77].

Despite the encouraging evidence of anti-IL6 agents in COVID-19, some treatment failures have been reported. According to phase III clinical trials of tocilizumab and sarilumab therapy in patients with severe pneumonia, IL-6 blockade may only be effective in the most severe conditions. Based on these observations, prevention of IL-6-mediated signaling may not be sufficient in critically ill patients with COVID-19-related CRS. Therefore, it may be necessary to interfere with other involved pathways in CRS. As such, combination therapy may be a promising approach for treating COVID-19. On the other hand, IL-6 has dramatic anti-inflammatory effects; thereby, there is a question about the administration of IL-6 antagonists as a therapeutic agent for reducing inflammation.

Several lines of evidence have shown that IL-6 has an important function in the early host immune reaction against infection. With this notion, IL-6 blockade may not be a successful strategy to cure the infection. On the other hand, the increased level of circulating IL-6 in patients with COVID-19 is not more than that in patients with autoimmune diseases, so it is unclear whether IL-6 is the contributory cytokine in COVID-19 pulmonary or not. In addition, it has been shown that using IL-6 inhibitors in autoimmune conditions increases the risk of severe infections and enhances the level of blood transaminase. Considering these points, the question arises regarding whether anti-IL-6 agents may have a detrimental impact on patients with COVID-19 [56].

As mentioned above, trans-signaling acts in a rather pro-inflammatory condition, mediated by mononuclear cell recruitment, prevention of T-cell apoptosis, and inhibiting the differentiation of regulatory T-cells. Taken together, it is assumed that blocking the trans-signaling pathway and maintaining the protective effect of the classical path may be a good strategy. In this context, it is suggested to hamper the uncontrolled inflammatory reaction of COVID-19 disease. With this background, other effective and safe strategies may be applied in severely affected patients.

### Other specific inhibitors for IL-6 trans-signaling cascade

As discussed above, the soluble form of IL-6R is generated by the enzymatic activity of ADAM17 (Fig. 1B). Given the broad expression of gp130 in most cells and subsequent activation by the IL-6/sIL-6R complex, a control mechanism is needed to avert IL-6- mediated inflammatory response under steady-state conditions. This can be partially achieved by a naturally occurring soluble gp (sgp) 130. As IL-6 is secreted, it binds to sIL-6R, and the forming complex will be neutralized by sgp130, as a buffering agent. At an elevated level of IL-6, the buffering capacity of sgp130 is not enough to overcome this; thereby, IL-6 mediated inflammatory responses are initiated. Addressing the detrimental activities of the trans-mediated pathway of IL-6, selective blockage of this pathway can prevent inflammatory responses.

The chimeric form of sgp130Fc can specifically hamper only pathological IL-6 trans-signaling pathways while retaining the protective effect and anti-inflammatory properties of the classic pathway (Fig. 1B) [78]. Sgp130Fc (Olamkicept) is a fusion protein of the extracellular domain of the gp130 receptor which is linked to the constant portion (Fc) of a human Ig G1 [79]. In this regard, sgp130Fc may be a selective inhibitor of IL-6 in COVID-19 infection and can be used as a specific substitute for tocilizumab. It has been reported that sgp130Fc administration in an animal model of polymicrobial sepsis could improve the survival rate up to 100%. Overall, the selective inhibition of only the pro-inflammatory (trans) cascade is a more reasonable choice than inhibiting both pathways. [80].

Furthermore, inhibition of ADAM-17-mediated cleavage may potentially suppress IL-6-trans-induced inflammation. A potent and highly selective inhibitor such as the A17 prodomain can be used in the severe form of COVID-19 with CRS [81, 82]. On the other hand, some evidence quarrel about the usage of anti-IL-6 agents for COVID-19 patients. It was declared that no benefit of anti-IL-6 was observed compared to standard therapy [83–85]. However, a crucial question arises regarding the therapeutic efficacy of IL-1 blockade, and whether IL-1 inhibition may confer a benefit over standard care management.

## The potential role of IL-1 in CRS

Growing evidence revealed the crucial role of IL-1 family members in inflammation. Among them, IL-1α and IL-1β have pro-inflammatory effects [86]. IL-1 as a pleiotropic cytokine has a substantial role in the pathogenesis of COVID-19. The higher levels of IL-1 can accumulate in the lungs of patients with COVID-19. IL-1 expresses in approximately all cell types, including epithelial cells, endothelial cells, and infiltrating myeloid cells within the damaged tissues, as observed in the lung of affected patients with COVID-19. Necrotic cell death in virus-mediated infection leads to the cell membrane rupture and subsequent release of IL-1α [87]. Both IL-1α and IL-1β bind to the biologically active receptor and inactive receptor. In order to activate the signaling pathway, the IL-1R must associate with the accessory protein [87]. In other words, IL-1α and IL-1β have the same pro-inflammatory effects through inflammatory-related signaling pathways. Along with this, several inflammatory-associated transcription factors are activated, for instance, NF-kB, activator protein-1, JNK, p38, mitogen-associated protein kinases (MAPKs), extracellular signal-regulated kinases (ERKs), and interferon-regulating genes [88].

IL-1α is expressed sufficiently by activated platelets, endothelial cells, and circulating monocytes during an inflammatory state [89]. Also, IL-1α is released from dead cells such as endothelial and epithelial cells, whereas IL-1β is released by immune cells, including neutrophils, macrophages, and infiltrating monocytes. Noteworthy, IL-I receptor antagonist (IL-1 Ra) can modulate and prevent excessive IL-1-mediated inflammatory responses via binding to IL-1 receptors. IL-1α and IL-1β are synthesized as precursor proteins; hence specific cellular proteases are needed to cleave and form mature cytokines for interacting with cell surface receptors. In addition to the mature form, the precursor of IL-1α is also active. However, the activity of this precursor is regulated by the decoy receptor through binding to IL-1α [90, 91].

It is well documented that the release of IL-1β mainly depends on the expression of the (NLR family pyrin domain containing 3) NLRP3 inflammasome, which controls the maturation of IL-1β. Published reports have suggested that NLRP3 inflammasome can recognize some RNA viruses [92]. As explained in previous sections, there is a life-threatening-associated cytokine storm in the advanced stage of COVID-19 disease, leading to diffuse alveolar epithelial and endothelial injury in the lung of affected patients [93]. Based on the IL-1-mediated inflammatory responses, it can be assumed that IL-1α-mediated inflammation is responsible for the development of COVID-19 pathogenesis. Once IL-1α is released from damaged epithelial cells, various pathological alterations are mediated through stimulation of several inflammatory cascades, sensing inflammatory myeloid cells, and activation of the inflammasome. Growing evidence showed that IL-1α plays a link between inflammatory reactions and the coagulation cascades. A local expression of IL-1α by endothelial cells promotes thrombosis by recruiting granulocytes to the lung[94]. Relying on this concept, the IL-1 blockade might avert thromboembolic events in COVID-19[95]. On the other hand, an IL-1 receptor antagonist (IL-1Ra) can limit inflammatory reactions and tissue damage during ARDS. Some clinical studies have declared that IL-1Ra is increased in the bronchoalveolar fluid in patients with ARDS, indicating the severe form of the disease [96]. With these backgrounds, overcoming IL-1-mediated hyper-inflammatory responses may have efficacy in patients with COVID-19. Accordingly, many ongoing clinical trials should be directed to evaluate the benefit of anti-inflammatory strategies in COVID-19.

### Anakinra

Anakinra is an antagonist of the IL-1 receptor that competitively blocks the IL-1-mediated activity through inhibition of IL-1 binding to its receptor (Fig. 1C). According to several clinical studies, specific blockade of IL-1α with monoclonal antibodies may have beneficial effects in alleviating the inflammatory storm in severe COVID-19 cases. Anakinra is a recombinant interleukin-1 receptor antagonist, which blocks the signaling pathways of both IL-1α and IL-1β. This immunomodulatory agent is approved for treating rheumatoid arthritis and autoinflammatory disease by administering at a daily dose of 100 mg daily via a subcutaneous injection [97]. A high-dose intravenous anakinra (5 mg/kg twice daily) improved systemic inflammation and respiratory complications in 29 COVID-19 patients with ARDS compared to standard care [98]. In a prospective, open-label, interventional trial, anakinra was evaluated in hospitalized patients with COVID-19 who required invasive mechanical ventilation. In addition to standard care, anakinra is administered at a dose of 100 mg twice daily for 3 days which is followed by 100 mg once daily for 7 days. Anakinra-treated patients (45 subjects) were compared with 24 historical controls. Anakinra treatment led to various events, including reducing invasive mechanical ventilation, improving respiratory dysfunction, and averting COVID-19-induced hyperinflammatory responses [99]. In another similar report, 52 patients with COVID-19 were treated with anakinra, and 44 historical patients served as controls. The encouraging results were obtained following anakinra therapy. Anakinra could prevent the requirement of mechanical ventilation and mortality in 25% of treated subjects compared to 37% of historical patients, conferring the effectiveness of this therapeutic intervention in the severe form of COVID-19 [100]. Another retrospective cohort study was conducted on 29 patients with COVID-19-induced ARDS with hyper inflammation (CRP ≥ 100 mg/L). Patients received a high dose of anakinra at a dose of 5 mg/kg twice daily. According to their results, high-dose anakinra dramatically improved survival rate compared to those receiving standard therapy. Moreover, the hyperinflammatory status was improved by reducing CRP levels [98].

### Canakinumab

As mentioned earlier, IL1-β has a key role in the pathogenesis of COVID-19; hence, blocking IL1-β-mediated activity is a reasonable therapeutic strategy (Fig. 1C). Canakinumab is a high-affinity, human monoclonal anti-IL-1β antibody specificity targeted IL-1β. Several studies have recently revealed the beneficial effects of canakinumab in patients suffering from severe COVID-19 [101, 102]. Canakinumab specifically inhibits IL-1 β and does not react with other IL-1 members [103]. Katia et al. et al. have surveyed the effect of canakinumab in COVID-19 disease. In 34 patients, 17 patients received a single dose of canakinumab with a dose of 300 mg, and the remaining were treated with standard therapy. According to their findings, canakinumab therapy considerably improved respiratory dysfunction and blood parameters compared to standard treatment. Besides, an improvement in the inflammation indices and oxygen flow was observed in canakinumab-treated groups [104].

Another similar study was conducted by Generali et al. on the impact of canakinumab in COVID-19 patients. Among 48 patients, 33 patients were treated with canakinumab, and 15 patients received only standard therapy as a control group. Canakinumab therapy was associated with earlier hospital discharge in treated patients (63%) compared to control groups. Moreover, this therapeutic agent could rapidly restore normal oxygen status and alleviate lung injury in affected patients. A substantial reduction in the count of white blood cells, platelets, and neutrophils was detected, accompanied by an increment in the lymphocyte count in canakinumab-treated patients. The serum level of CRP was declined, too.

Based on the benefits reported here and considering restoring normal oxygen status, decreasing the need for mechanical ventilation, and improving the clinical symptoms in infected patients, canakinumab may be a useful therapeutic intervention in patients with COVID-19 [105]. Despite the remarkable properties of canakinumab, some reports have stated hematological alterations such as leukopenia, thrombocytopenia, and neutropenia in treated patients. An elevated level of liver enzymes has been detected, too [106]. Collectively, it is inferred that the administration of canakinumab can ameliorate CRS and dampen the clinical complications of COVID-19.

### Comparison of anti-IL-6 and IL-1 antagonists

Comparing IL-1 and IL-6 inhibitors were reported in an observational study in Italy COVID-19 patients with severe respiratory dysfunction and hyperinflammation. Of 392 patients, 62 received anakinra, 55 were treated with IL-6 inhibitors such as Tocilizumab and Sarilumab, and the remaining did not receive any cytokine inhibitor. Reducing the mortality rate was observed in patients who revived the only anakinra. IL-6 inhibitors showed efficacy in affected patients with higher levels of CRP. The reason behind this contradiction can be explained by IL-6, which stimulates the liver to produce CRP. Besides, both IL-1 and IL-6 inhibitors have the benefit in affected subjects with low levels of lactate dehydrogenase [107].

Based on this evidence, the efficacy of IL-1 blockade may be related to endotheliopathy of COVID-19 to secrete IL-1α. Given the upstream role of IL-1 in the IL-6 signaling pathway, IL-1 blocking may prevent IL-6 activity. Such a relationship would be significant in developing IL-1 antagonists for patients with COVID-19 and CRS [108]. Furthermore, the superior safety of anakinra is attributed to lower opportunistic infections and short half-life through the rapid clearance compared to the prolonged half-life of IL-6 inhibitors (2–3 weeks) [109]. Despite encouraging benefits, IL-1 blockade has been accompanied by safety and warning issues such as an increased risk of serious infections. Another concern with anakinra is neutropenia, which requires an evaluation of neutrophil counts before starting treatment [106].

## The role of IL-17 cytokine in COVID-19 infection

It is most well-known that T helper (Th) cells have a critical role in the adaptive immune system during viral infections. Indeed, following the recognition of the virus by antigen-presenting cells (APCs), dendritic cells (DCs) initiate cytokine release and form a microenvironment to evoke T cell responses. Activation of Th17 cells can lead to the secretion of multiple inflammatory cytokines. It has been reported that naïve CD4^+^ T cells can differentiate into Th17 cells via activation of Th cells in the presence of IL-6, IL-1β, IL-23, and TGF-β [110]. According to current literature, alveolar macrophages such as APCs can release IL-6, IL-23, and many other cytokines in COVID-19. The binding of IL-6 and IL-23 to the corresponding receptors can cause polarization and maturation of naïve CD4^+^ T cells toward Th17 cells [111]. Th1 cells primarily regulate the adaptive immune response through cytokine production, and cytotoxic T-lymphocytes (CTLs), known as CD8^+^ T cells, kill virus-infected cells [112].

IL-17 is a member of pro-inflammatory cytokines secreted by Th17 cells, which has a crucial role in the recruitment of monocytes and neutrophils to the site of infection. The IL-17 family includes 6 members: IL-17A (known as IL-17) and related family members, including IL-17B, IL-17C, IL-17D, IL-17E (known as IL-25), and IL-17F. IL-17A is the most critical member of this family, known as IL-17 (Fig. 1D) [113]. IL17 can exacerbate inflammatory reactions by activating downstream cytokines, such as IL-1, IL-6, IL-8, TNF-α, and MCP-1 [114]. Moreover, IL-17 can induce secretion of G-CSF, GM-CSF, and various chemokine ligands, for instance, CXCL1, CXCL2, and CXCL8. IL-17A also induces IP-10, MIP-1α/β, and multiple matrix metalloproteinases (MMPs) [115]. In addition, there is a link between IL-17 and IL-6 mediated activity in viral infections. In this regard, Hou et al. reported that overproduction of IL-6 levels could enhance IL-17-producing Th17 cells. In this case, IL-17 and IL-6 synergistically promote viral persistence by hindering host defense [116]. Noteworthy, the increased Th17 cells and IL-17 responses were reported in the severe form of COVID-19 patients. Upregulation of this cytokine is almost associated with lung pathology and ARDS and can injure other organs such as the heart, liver, and kidney [117].

Several evidence reported that the severity of COVID-19 is strongly correlated with IL-17 -induced inflammation, beyond other pro-inflammatory cytokines. ICU admitted COVID-19 patients have elevated levels of Th17 cells and worsened clinical manifestations in comparison to non-ICU patients. This is possible due to the excessive production of IL-17A and other pro-inflammatory cytokines [30, 118]. Altogether, it is hypothesized that IL-17 blockade is an immunologically plausible approach to improve the aberrant immune responses in COVID-19 and hinder ARDS-associated mortality [119].

It has been well established that the JAK-STAT pathway mediates the differentiation of Th17 cells. The signals from IL-6 and IL-23 can cause TH17 cell polarization from naïve CD4^+^. Both IL-6 and IL-23 activate STAT3 through JAK2. Accordingly, targeting JAK with specific inhibitors could be a possible approach to restrict Th17 cells-induced hyperinflammation. Evidence instantiates that JAK2 inhibitors such as fedratinib could decrease IL-17 secretion in murine Th17 cells [120].

Also, another study found that Fedratinib has efficacy in reducing the expression level of IL-17 by murine Th17 cells. In addition to its effect on IL-17, fedratinib may also inhibit GM-CSF activity, as GM-CSF can interact with JAK2 to mediate signaling. It can be implied that fedratinib can prevent the production of Th17 signature cytokines. Thus, this intervention would be a promising approach to alleviate the deteriorating effects of Th17-related inflammatory responses in COVID-19 [121].

There are multiple antibodies against Th17, including anti-IL-17, and anti-IL-17R; however, these antibody-based therapies have limited beneficial effects. To date, three IL-17 blocking agents are available, including ixekizumab, secukinumab, and brodalumab. Ixekizumab and secukinumab are both monoclonal IgG1 antibodies that specifically target IL-17A, with a similar mechanism of action. Both have high efficacy, tolerability, and a good safety profile, without decreasing the lymphocyte count. In patients with severe COVID-19, the lymphocyte count was considerably reduced, so these two antagonists can be considered reasonable interventions in COVID-19 patients. Brodalumab is a recombinant human monoclonal antibody against anti-IL-17 receptor A (IL-17RA). This therapeutic option can completely block T-17- mediated pathways [119]. COVID-19 is characterized by an immune dysregulation rather than a viral load, which leads to abnormal production of pro-inflammatory cytokines by alveolar macrophages. In this case, ARDS is the most common outcome of the exuberant infiltration of inflammatory cells responsible for COVID-19 infection and subsequent secretion of pro-inflammatory cytokines [122].

## The role of TNF-α in COVID-19 infection

TNF-α is also associated with bronchial hyperresponsiveness. TNF-α is linked with the reduction of airway calibre and enhanced neutrophilia in the epithelium of the respiratory tract. TNF-α can directly deteriorate the respiratory epithelium by producing inflammatory cytokines such as GM-CSF, IL-8, and intercellular adhesion molecules (ICAMs). Besides, TNF-α can induce the release of MMP-9 by neutrophils. All these events lead to irreversible alteration via pulmonary fibrosis [123].

TNF-α is detected in the blood and tissues of patients with COVID-19. It is well established that TNF-α has a fundamental role in almost acute inflammatory responses as an amplifier and even as a coordinator of inflammation. TNF-α is mainly produced by monocytes and macrophages. In addition, B-cells, T-cells, and fibroblasts can synthesize TNF-α. TNF-α exerts its activity by interacting and activating with two receptors, TNF receptor (TNFR) 1 and TNFR2 in order to transduce signals [124]. TNF is initially synthesized as a bioactive transmembrane precursor protein with a molecular weight of 26 kDa. To release the soluble active TNF protein (17 kDa), it can undergo proteolytic cleavage by the TNF-α-converting enzyme, ADAM17. TNFR1 signaling is activated by two forms of TNF receptors as soluble (sTNF) and transmembrane (tmTNF), while TNFR2 is activated by tmTNF [123].

Given the profound role of excessive TNF in the development, pathogenesis, and poor outcome of COVID-19, blockade of TNF offers a clinically effective intervention in this regard. Importantly, blocking of TNF- mediated inflammatory response leads to a rapid decline in the level of IL-6 and IL-1 in individuals with active inflammation. The data from inflammatory bowel disease (IBD) patients with COVID-19 revealed that among 116 patients who received anti-TNF therapy, 99 patients recovered without hospital admission. Besides, affected patients on anti-TNF treatment face a better outcome [125].

Five specific inhibitors against TNF were initially approved for clinical applications, including infliximab, adalimumab, golimumab, certolizumab, and etanercept. Etanercept is a TNF antagonist that binds to the members of the lymphotoxin (LT) family. Indeed, these anti-TNF agents have been utilized for many years in severe autoimmune and inflammatory disease cases. The mechanism of actions of these antagonists is based on binding to their cognate ligands such as sTNF or tmTNF, and for etanercept, by binding to LTα3 and LTα2β and blocking downstream cascades. In other words, all five antagonists obstruct interaction between TNF and corresponding receptors on expressed cells. More details about these antagonists are depicted in Table 1 [126]. In addition, it has been reported that IL-17A and TNF-α have a substantial role in lung damage of covid-19 disease with obesity [127].

**Table 1:** Anti TNF agents

	Structure	Cognate ligands	Half life	Dosing	Frequency
Infliximab (Remicade®)	Chimeric (mouse and human)/ whole mAB against TNF	sTNF, tmTNF	8–10	Intravenous	Very 8 weeks following loading at 0, 2 and 6 weeks
Adalimumab (Humira®)	Human whole mAb against TNF	sTNF, tmTNF	10–14	Subcutaneous	Every 2 weeks following initial loading
Golimumab (Simponi®)	Human whole mAb against TNF	sTNF, tmTNF	12 ± 3	Subcutaneous	Monthly following initial loading
Certolizumab	Humanized PEGylated Fab fragment of a mAb against TNF	sTNF, tmTNF	3	Subcutaneous	Every 2 weeks following initial loading
Etanercept# (Enbrel®)	TNFR2 fused to IgG1 Fc	sTNF, tmTNF, LTα3	14	Subcutaneous	Weekly/twice Weekly

*LTα3* trimeric lymphotoxin α, *sTNF* soluble TNF, *tmTNF* transmembrane TNF, *mAb* monoclonal antibody

## Conclusion and perspectives

Dysregulated acquired immune system, and hyperinflammatory innate immune responses may be responsible for cytokine storm in COVID-19. Herein, we discussed potential mechanisms behind the COVID-19-induced CRS and then summarized possible therapeutic approaches. Indeed, the CRS is closely associated with fatal outcomes in critically severe COVID-19 patients. The management of the cytokine storm by cytokine antagonists and immunomodulatory agents may improve the survival rate of the infected patients. In this regard, targeting inflammatory cytokines can benefit patients and enhance the therapeutic efficacy of anti-viral therapy in COVID-19 patients.

On the other hand, targeting one inflammatory signaling pathway might stimulate downstream compensatory immune responses due to the complexity of the inflammatory network. Therefore, balancing the risk versus benefit of anti-inflammatory drugs should be considered. The data presented here and the authors' perspective have identified that antibodies targeting inflammatory cytokines remain an attractive therapeutic approach; thereby, combination therapy of inflammatory inhibitors and other COVID-19 modalities may have a better impact than alone. Highly approved evidence is required to understand the underlying mechanism of cytokine storms in COVID-19. Further studies are needed to elucidate the importance of anti-inflammatory interventions to curb hyperinflammation.

## Data Availability

Not applicable.

## Abbreviations

SARS-CoV-2: Severe acute respiratory syndrome coronavirus-2
COVID-19: Coronavirus disease 2019
ARDS: Acute respiratory distress syndrome
CRS: Cytokine release syndrome
IL: Interleukin
TNF: Tumor necrosis factor
ICU: Intensive care unit
INF: Interferon
Ig: Immunoglobulin
MCP: Monocyte chemoattractant protein
FcR: Fc receptor
ACE2: Angiotensin-converting enzyme 2
BBB: Blood brain barrier
GM-CSF: Granulocyte–macrophage-colony stimulating factor
Th: T helper
NF-kB: Nuclear factor kappa light chain enhancer of activated B cells
IFN-γ: Interferon-gamma
G-CSF: Granulocyte colony-stimulating factor
IP: Inducible protein
MIP-1α: Macrophage inflammatory protein
M-CSF: Macrophage colony-stimulating factor
mbIL-6R: Membrane-bound IL-6 receptor
JAK/STAT: Janus kinase/signal transducer and activator of transcription
VEGF: Vascular endothelial growth factor
ADAM17: A disintegrin and metalloprotease 17
TGF-β2: Transforming growth factor-beta 2
RAS-RAF: Rat sarcoma-rapidly accelerated fibrosarcoma
PI3KB: Phosphatidylinositol 3-kinase/protein kinase B
TCZ: Tocilizumab
FDA: Food and drug administration
RA: Rheumatoid arthritis
RCTs: Randomized controlled trials
CYP3A4: Cytochrome P450 3A4
CRP: C-reactive protein
MCD: Multicentric castleman disease
sgp 130: Soluble gp130
MAPKs: Mitogen-associated protein kinases
ERKs: Extracellular signal-regulated kinases
APCs: Antigen-presenting cells
DCs: Dendritic cells
CTLs: Cytotoxic T-lymphocytes
MMPs: Matrix metalloproteinases
IL-17RA: Anti-IL-17 receptor A
ICAMs: Intercellular adhesion molecules
TNFR: TNF receptor
IBD: Inflammatory bowel disease
LT: Lymphotoxin
